# The release of GLP‐1 from gut L cells is inhibited by low extracellular pH

**DOI:** 10.1002/oby.24125

**Published:** 2024-09-05

**Authors:** Philippa Garbutt, Malgorzata Cyranka, Johanna Michl, Yuko Maejima, Natascia Vedovato, Kenju Shimomura, Pawel Swietach, Heidi de Wet

**Affiliations:** ^1^ Department of Physiology, Anatomy and Genetics University of Oxford Oxford UK; ^2^ Department of Bioregulation and Pharmacological Medicine Fukushima Medical University School of Medicine Fukushima Japan

## Abstract

**Objective:**

The intestinal luminal pH profile varies from stomach to rectum and becomes disrupted in diseases. However, little is known about the pH dependence of incretin hormone secretion, with most in vitro studies having failed to consider this modulatory factor or having used nonphysiological buffer systems. Here, we report the extracellular pH (pHe) dependence of glucagon‐like peptide‐1 (GLP‐1) exocytosis from L cells.

**Methods:**

The pHe dependence of GLP‐1 release from GLUTag cells and murine ex vivo primary gut cultures was detected by ELISA. GLP‐1 release was measured over a range of pHe under a physiological (CO_2_/HCO_3_^−^) buffering regime and in its absence (HEPES buffer). The relationship between intracellular pH (pHi) and pHe was mapped given that at least some component of pH sensitivity is likely to be intracellular.

**Results:**

GLP‐1 secretion from L cells was pHe‐dependent and stimulated under alkaline conditions. In the absence of glucose or extracellular calcium, secretion remained at a pHe‐insensitive baseline. pHi followed changes in pHe, but the relationship was offset to more alkaline levels in the absence of CO_2_/HCO_3_^−^ buffer and became shallower if [Cl^−^] changes that normally accompany [HCO_3_^−^] changes were compensated iso‐osmotically with gluconate.

**Conclusions:**

GLP‐1 secretion is sensitive to pHe and the buffer present. Exploiting this mechanism therapeutically may benefit patients with obesity.

**Study Importance**

**What is already known?**

- To our knowledge, this is the first study to report in detail the effect of extracellular pH (pHe) on glucagon‐like peptide‐1 (GLP‐1) exocytosis rates and the effect of pHe on intracellular pH (pHi) in enteroendocrine cells (EECs).

**What does this study add?**

- GLP‐1 secretion rates in a nonphysiological HCO_3_^−^‐free buffer such as HEPES are considerably different from those in a CO_2_/HCO_3_^−^ buffer, and an alkaline offset of 0.138±0.025 pH units was observed when the physiological buffer (CO_2_/HCO_3_^−^) was replaced with HEPES buffer. Cl^−^ ions impact the pHe–pHi relationship of EECs, implicating a role for anion exchangers in fine‐tuning the pH response of EECs to a changing extracellular acid–base milieu.

**How might these results change the direction of research or the focus of clinical practice?**

- The luminal pH of the small intestine and colon varies and is altered in disease. How luminal pH, and the disruption thereof, affects GLP‐1 release is an important factor to consider when developing effective strategies to treat obesity.

## INTRODUCTION

Radiotelemetry in healthy human volunteers revealed dynamic and substantial variation of luminal pH throughout the gastrointestinal tract, with a range from pH 5 up to pH 8 [1, 2, 3]. However, disturbances from this pattern, i.e., notably, a reduction in colonic lumen pH, were described in patients with inflammatory bowel disease [4]. Recent studies have reported an association between alterations of the gut pH and diabetes and obesity [5, 6]. Specifically, a lower pH was observed in the small intestine of individuals with obesity compared with those with normal weight in the fasted and fed states [5]. A substantial body of data is available on the links among obesity, pH, gut microbiota, the effects of weight loss surgeries such as Roux‐en‐Y gastric bypass, and altered intestinal physiology [7, 8, 9, 10]. The stimulation secretion coupling of the two incretin hormones, i.e., glucagon‐like peptide‐1 (GLP‐1) and glucose‐dependent insulinotropic polypeptide (GIP), secreted by the enteroendocrine cells (EECs) of the intestines, is well known to be affected by metabolic disease [11]. GLP‐1 receptor agonists are now routinely used to treat obesity and type 2 diabetes [12], but the impact of intestinal pH on enteroendocrine EEC activity, and specifically GLP‐1 secretion, remains unknown.

The incretin hormone GLP‐1 is a good model to gauge the activity of EECs because stimulation secretion coupling of this incretin hormone is regulated by a wide range of factors, many of which are impacted by metabolic dysregulation [13, 14]. Currently, there is little information regarding the pH dependence of GLP‐1 secretion from enteroendocrine L cells. Because pH is intricately linked to buffering, there are equally no data available on how different experimental buffering regimes that are typically used in the laboratory affect GLP‐1 release. Here, we report the pH dependence of GLP‐1 exocytosis from L cells in the presence of a physiological bicarbonate buffering system.

## METHODS

### Cell culture of GLUTag cells and isolation and culture of gut primary cells from C57BL/6N wild‐type mice

GLUTag cells were a kind gift from Professor Daniel J. Drucker (University of Toronto). Standardized cell culture protocols for GLUTag cells and gut primary cell cultures have been described in detail in Cyranka et al. [15].

### GLP‐1 secretion assays

Active GLP‐1 secretion from GLUTag cells was measured by fluorescence resonance energy transfer‐based enzyme‐linked immunosorbent assay (ELISA) for active GLP‐1 (62GLPPEG, Cisbio) or total GLP‐1 (catalog #EZGLP1T‐36 K, Millipore) as described previously [15] and detailed in online Supporting Information.

### HEPES‐ and CO_2_ /HCO_3_ ^−^‐buffered media

A detailed description of the preparation and composition of all buffers is given in Tables S1 through S3 [16].

### Intracellular pH measurements

Intracellular pH (pHi) was measured using carboxy SNARF‐1 (cSNARF1), as detailed in online Supporting Information and shown in Figure S6. Analysis of the population distribution of pH data was performed using a MATLAB script [16].

### Statistical analysis

Data are presented as mean (SEM) with individual data points shown. Data were analyzed using one‐ or two‐way ANOVA with Tukey post hoc tests, as indicated in the figure captions. *P* < 0.05 was regarded as statistically significant. Statistical analysis was performed using Prism 6 (Graphpad Software).

## RESULTS

### GLP‐1 secretion from GLUTag cells and murine ex vivo primary gut cultures is sensitive to extracellular pH

**FIGURE 1:**
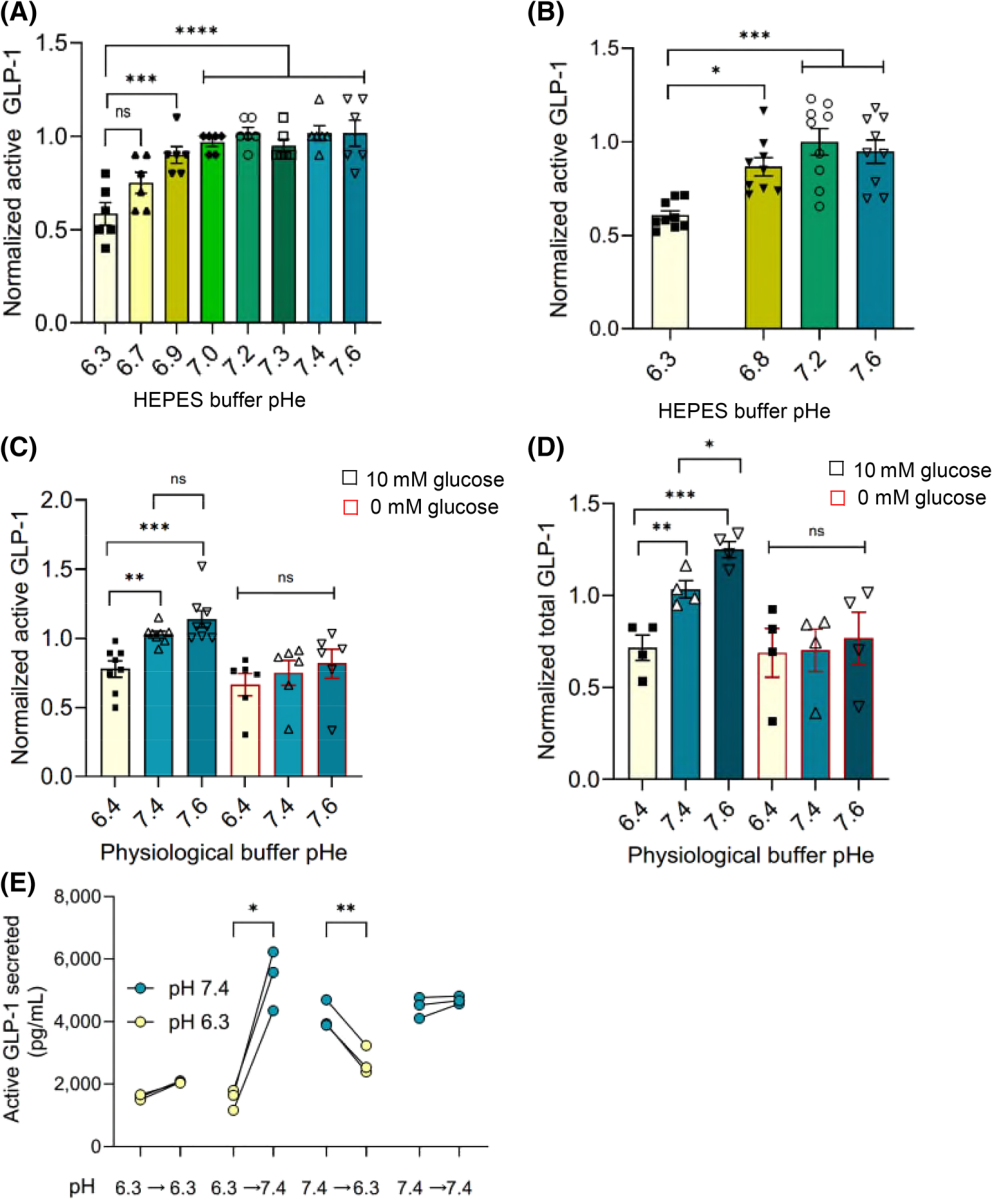
Glucagon‐like peptide‐1 (GLP‐1) secretion from GLUTag cells and primary L cells is pH‐dependent. (A) pH dependence of active GLP‐1 secretion from GLUTag cells in HEPES buffer, 10mM glucose. Data were normalized to secretion levels at pH 7.4. Data are shown as mean ± SEM, *n* = 6–8. All values recorded for pH 6.9 and above are significantly different from pH 6.3. (B) pH dependence of active GLP‐1 secretion from murine mixed primary small intestinal cultures in HEPES buffer. GLP‐1 secretion was measured from mixed primary small intestinal cultures isolated from C57BL/6N wild‐type mice. Data are shown as mean ± SEM for *n* = 5–6 separate primary culture preparations taken from two different mice. For panels A and B, data were normalized to secretion levels at pH 7.2. (C) pH dependence of active GLP‐1 secretion from GLUTag cells in physiological bicarbonate buffer, 10mM glucose or no glucose. Data are shown as mean ± SEM, *n* = 6–8. (D) pH dependence of total GLP‐1 secretion from GLUTag cells in physiological bicarbonate buffer, 10mM glucose or no glucose. Data are shown as mean ± SEM, *n* = 6. For panels C and D, data were normalized to secretion levels at pH 7.4 in 10mM glucose. (E) The pH effect on GLP‐1 secretion from GLUTag cells in HEPES buffer is reversible. GLUTag cells were incubated for 1 h at pH 6.3 or pH 7.4, after which the solution was either switched or replaced with same for a further hour before analysis, *n* = 3 per paired experiment. *p < 0.05, **p < 0.01, ***p < 0.001, ^****^ *p* < 0.0001, and “ns” denotes not significant. Data were analyzed by one‐way ANOVA using multiple comparisons with Tukey post hoc test. pHe, extracellular pH. [Color figure can be viewed at wileyonlinelibrary.com]

GLP‐1 secretion from both GLUTag cells (Figure 1A) and ex vivo primary gut cultures (Figure 1B) was reduced at low pH in a nonphysiological HEPES buffering system. Thus, raising extracellular pH (pHe) from 6.3 to 7.6 doubled GLP‐1 secretion from GLUTag cells and ex vivo primary gut cultures (1.8 [0.3]‐fold and 1.9 [0.2]‐fold, respectively). A similar trend was observed when GLP‐1 secretion was measured in the presence of a physiological CO_2_/HCO_3_^−^ buffering system for both active GLP‐1 (Figure 1C) and total GLP‐1 (Figure 1D), i.e., 1.4 (0.05)‐fold and 1.8 (0.1)‐fold upregulated, respectively. The pH dependence of GLP‐1 secretion manifested only in the presence of glucose (Figure 1C,D). Data are also shown as GLP‐1 released in picograms per milliliter in Figure S1A–D. Interestingly, this pH dependence of GLP‐1 secretion was rapidly reversible, indicating a dynamic process (Figure 1E). The pH‐dependent changes observed were not a result of altered ELISA detection efficiencies (Figure S2A,B), degradation of secreted GLP‐1 or GLP‐1 peptide stability (Figure S3A,B), or altered cell viability at low pH (Figure S4).

### The rate of GLP‐1 release is pHe‐dependent

**FIGURE 2:**
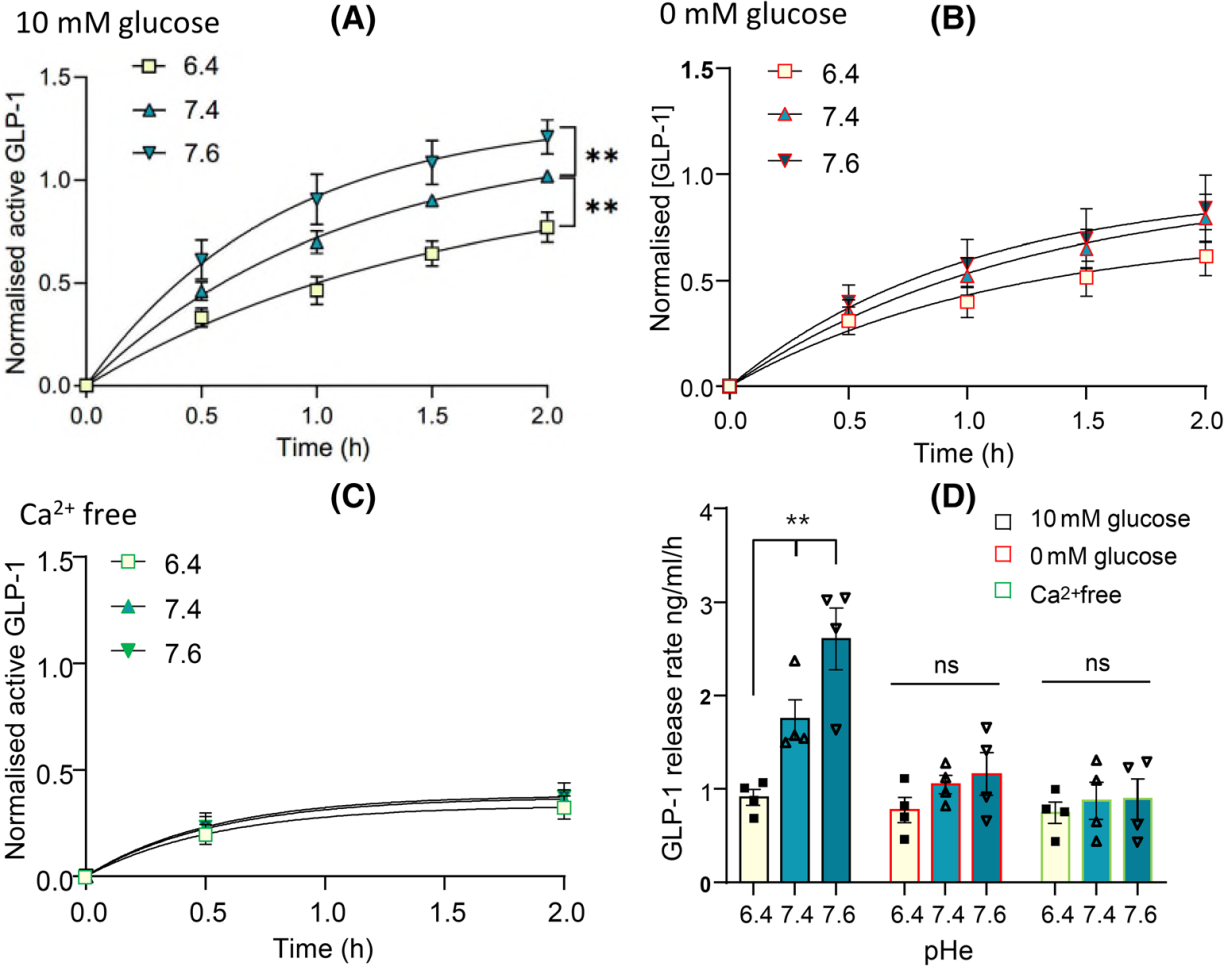
Glucagon‐like peptide‐1 (GLP‐1) release rate is pH‐dependent. Extracellular pH (pHe) dependence of the rate of active GLP‐1 secretion from GLUTag cells in physiological buffer in the presence of (A) 10mM glucose (two‐way ANOVA; pH: *p* < 0.01, time: *p* < 0.001, interaction: *p* < 0.001), (B) no glucose (two‐way ANOVA; pH: *p* = ns, time: *p* < 0.001, interaction: *p* = ns), and (C) in the absence of Ca^2+^ (two‐way ANOVA; pH: *p* = ns, time: *p* < 0.001, interaction: *p* = ns). The time course of GLP‐1 secretion was plotted with the best fit line to the exponential equation *y* = a*(1‐exp[−x/b]). Data of panels A, B, and C were normalized to the total average secretion levels at the 2‐h time point in 10mM glucose. (D) Initial rate of GLP‐1 secretion in nanograms per milliliters per hour at pHe 6.4, 7.4, and 7.6. Each point/bar represents mean ± SEM from *n* = 4. Significant values are depicted as **p < 0.01, “ns” denotes nonsignificant, and data are analyzed by one‐way ANOVA with Tukey post hoc test. [Color figure can be viewed at wileyonlinelibrary.com]

The effect of pHe on GLP‐1 levels could arise from a pHe sensitivity of secretion rate or negative feedback of GLP‐1 on its secretion that emerges only at low pHe. These alternative mechanisms can be distinguished by measuring the time course of GLP‐1 secretion and determining the initial rate. To test this, a longitudinal study using GLUTag cells obtained the time course of GLP‐1 levels over the first 2 h of culture. The time course was fitted to exponential curves to obtain the initial rate and assess its pHe sensitivity in the presence (Figure 2A) and absence of glucose (Figure 2B), as well as under calcium‐free conditions (Figure 2C). Initial GLP‐1 secretion rates increased more than twofold from 0.9 (0.08) ng/mL/h to 2.6 (0.3) ng/mL/h at pH 6.3 to pH 7.6, respectively (Figure 2D), but became pHe‐insensitive under baseline conditions of glucose‐ or calcium‐free media. Thus, the pHe sensitivity emerges from the onset of GLP‐1 secretion rather than through delayed feedback. Data are also shown as GLP‐1 released in picograms per milliliter in Figure S5A–D.

### The relationship between pHi and pHe depends on the buffering regime

**FIGURE 3:**
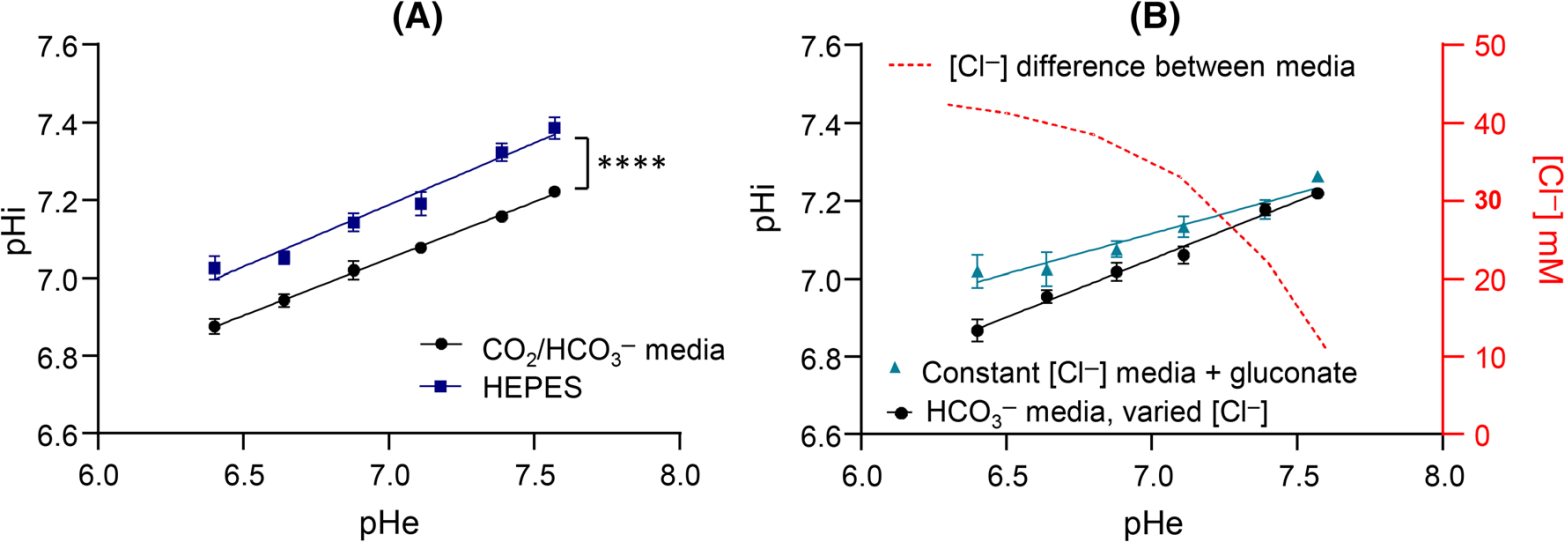
GLUTag cell intercellular pH (pHi) in response to changes in extracellular pH (pHe) is different in the presence of HEPES or physiological buffer system. (A) pHi relative to pHe measurements across pHe 6.4 to 7.6 in the presence of 10mM glucose at 37°C and either 5% CO_2_ in bicarbonate buffer or 0% CO_2_ in HEPES‐buffered media (mean ± SEM, *n* = 7, two‐way ANOVA; pHe: *p* < 0.0001, buffer: *p* < 0.0001, interaction: *p* < 0.0001). ****p < 0.0001. (B) pHi regulation in GLUTag cells in CO_2_/ HCO_3_^−^‐based media in the presence of increased [Cl^−^] to compensate for osmolarity (black circles) or where gluconate ions replace osmolarity‐regulating excess Cl^−^ ions (blue triangles). The red line indicates the difference in extracellular [Cl^−^] between the two different media (mean ± SEM, *n* = 5, two‐way ANOVA; pHe: *p* < 0.001, buffer: *p* < 0.001, interaction: *p* < 0.001. [Color figure can be viewed at wileyonlinelibrary.com]

The effect that changes in pHe have on pHi was imaged in SNARF‐1‐loaded GLUTag cells (Figure 3A). Extracellular acidification reduced cytoplasmic pH, consistent with low pHe inhibiting acid‐extruders and stimulating acid‐loaders [17]. However, an alkaline offset (0.138 [0.025] pH unit) was observed when the physiological buffer (CO_2_/HCO_3_^−^) was replaced with HEPES buffer. This indicates substrate‐mediated activation of HCO_3_^−^‐dependent transporters, which tend to reduce pHi. At constant CO_2_, a reduction in [HCO_3_^−^] to lower pH is coupled to a rise in [Cl^−^] as NaCl is added to maintain osmolarity (buffer composition stipulated in Table S1). Thus, an increase in [Cl] at low pHe may affect pHi. To test this, a second round of experiments kept [Cl^−^] constant by compensating for changes in [HCO_3_^−^] with changes in gluconate, a membrane‐impermeable anion. This resulted in a shallower pHi–pHe relationship (Figure 3B). The transmembrane [Cl^−^] gradient drives exchange with HCO_3_^−^ by transporters such as chloride/bicarbonate anion exchangers of the solute carrier 4 (SLC4) or SLC26 families. Normally, these act as acid‐loaders driven by Cl^−^ influx. When extracellular [Cl^−^] is kept constant experimentally, anion exchange (acid‐loading) activity is reduced, which explains why the pHi–pHe gradient is significantly shallower (0.2975 vs. 0.2068; *p* = 0.0174). Differences in steady‐state pHi between standard conditions and constant [Cl^−^] were significant at pHe 6.4 (pHi 7.02 [0.04] and 6.87 [0.03], respectively; *p* = 0.0013), coinciding with the largest difference in media [Cl^−^]. Noticeably, at pHe 6.4, the increase in pHi in constant [Cl^−^] media is comparable with the effect of removing HCO_3_^−^. Overall, the results indicate that anion (Cl^−^/HCO_3_^−^) exchangers are likely to influence the pHi–pHe relationship in GLUTag cells and, by extension, in EECs. However, because a pH dependence of GLP‐1 secretion was also observed in the presence of HEPES buffer, HCO_3_^−^ independent mechanisms are also present.

## DISCUSSION

Many cells in the body are exposed to a constant pHe because of the clamping effect of continuous blood flow. However, some cells will be exposed to large and dynamic changes in pHe, such as the epithelia and EECs of the gut. Herein, we describe how pHe affects GLP‐1 secretion as a proof‐of‐concept finding that luminal pH of the gut can affect EEC function. Some pH sensors are at the surface of cells, but a large capacity for pH sensitivity is held on intracellular sites, where essentially all proteins show a degree of pH sensitivity [17]. Therefore, it is important to test how pHe affects pHi in order to postulate mechanisms that may or may not implicate exofacial sensors. We found that low pHe, at 6.4, reduces GLP‐1 secretion rate to baseline levels normally observed in the absence of glucose or calcium. This pH dependence was seen in GLUTag, as well as murine mixed primary small intestinal cultures. We also mapped the pHe–pHi relationship, implicating a role for anion exchangers in fine‐tuning the cell's pH response to a changing extracellular acid–base milieu. Indeed, expression of the chloride/bicarbonate anion exchangers AE2 and AE3 isoforms (*SLC4A2* and *SLC4A3*, respectively) in GLUTag cells was confirmed from RNA sequencing (RNAseq) analysis [18, 19], Gene Expression Omnibus (GEO) accession number GSE193866.

However, there are limitations to the work presented here. First, the precise sensor(s) that underpin the observed inhibitory effect of pHe on GLP‐1 exocytosis remains to be identified but could be a combination of intra‐ and extracellular mechanisms. The alkaline shift of pHi in response to changes in pHe in both the absence of CO_3_^−^ and constant [Cl^−^] suggests the involvement of AE2 and AE3, but this remains to be confirmed [20]. Understanding these mechanisms may provide new therapeutic routes of intervention via modulating GLP‐1 secretion in patients with obesity. Second, the cells used in this study are not polarized, the assumption being that, under physiological conditions, the apical surface of the cell would be exposed to luminal fluctuations in pH (pHe), whereas the basolateral surface would not. This would necessitate pHe‐sensitive machinery to localize to the apical membrane of polarized EECs.

## CONCLUSION

It is known that pathology can lower intestinal pH. Herein, we show for the first time, to our knowledge, that the secretion rate of GLP‐1 from L cells is decreased at low pHe, a mechanism that is conserved in both GLUTag cells and ex vivo primary gut cultures. Furthermore, results from experiments conducted in HCO_3_^−^‐free media should be interpreted with caution.

## FUNDING INFORMATION

Malgorzata Cyranka and Heide de Wet were supported by the Biotechnology and Biological Sciences Research Council (BBSRC; BB/P020666/1).

## CONFLICT OF INTEREST STATEMENT

The authors declared no conflict of interest.

## Supporting information


**FIGURE S1:** GLP‐1 secretion (pg/mL) from GLUTag cells and primary L‐cells is pH dependent.
**FIGURE S2:** The observed pH effect is not a result of altered ELISA (Cisbio) detection efficiencies.
**FIGURE S3:** Degradation of the secreted GLP‐1 and GLP‐1 peptide stability is not affected by pH.
**FIGURE S4:** Cell viability assays.
**FIGURE S5:** GLP‐1 release rate is pH dependent.
**FIGURE S6:** (A) Representative histograms of pHi measured at pHo 6.4, 7.1 and 7.6 in 10mM glucose.

**DATA S1:** Supporting Information.
Additional supporting information can be found online in the Supporting Information section at the end of this article.

## References

[1] Koziolek M , Grimm M , Becker D , et al. Investigation of pH and temperature profiles in the GI tract of fasted human subjects using the IntelliCap system. J Pharm Sci. 2015;104:2855‐2863. 25411065 10.1002/jps.24274

[2] Maurer JM , Schellekens RC , van Rieke HM , et al. Gastrointestinal pH and transit time profiling in healthy volunteers using the IntelliCap system confirms Ileo‐colonic release of ColoPulse tablets. PLoS One. 2015;10(7):e0129076. 26177019 10.1371/journal.pone.0129076 PMC4503763

[3] Khutoryanskiy VV . Supramolecular materials: longer and safer gastric residence. Nat Mater. 2015;14(10):963‐964. 26395936 10.1038/nmat4432

[4] Nugent SG , Kumar D , Rampton DS , Evans DF . Intestinal luminal pH in inflammatory bowel disease: possible determinants and implications for therapy with aminosalicylates and other drugs. Gut. 2001;48:571‐577. 11247905 10.1136/gut.48.4.571 PMC1728243

[5] Steenackers N , Wauters L , van der Schueren B , et al. Effect of obesity on gastrointestinal transit, pressure and pH using a wireless motility capsule. Eur J Pharm Biopharm. 2021;167:1‐8. 34273543 10.1016/j.ejpb.2021.07.002

[6] Anhe FF , Varin TV , Schertzer JD , Marette A . The gut microbiota as a mediator of metabolic benefits after bariatric surgery. Can J Diabetes. 2017;41:439‐447. 28552651 10.1016/j.jcjd.2017.02.002

[7] Steenackers N , Vanuytsel T , Augustijns P , et al. Adaptations in gastrointestinal physiology after sleeve gastrectomy and Roux‐en‐Y gastric bypass. Lancet Gastroenterol Hepatol. 2021;6(3):225‐237. 33581761 10.1016/S2468-1253(20)30302-2

[8] Lin K , Zhu L , Yang L . Gut and obesity/metabolic disease: focus on microbiota metabolites. MedComm (2020). 2022;3:e171. 10.1002/mco2.171 PMC9437302 36092861

[9] Shalon D , Culver RN , Grembi JA , et al. Profiling the human intestinal environment under physiological conditions. Nature. 2023;617(7961):581‐591. 37165188 10.1038/s41586-023-05989-7 PMC10191855

[10] Yamamura R , Inoue KY , Nishino K , Yamasaki S . Intestinal and fecal pH in human health. Front Microbiomes. 2023;2:1192316. 10.3389/frmbi.2023.1192316 PMC12993532 41853365

[11] Nauck MA , Meier JJ . The incretin effect in healthy individuals and those with type 2 diabetes: physiology, pathophysiology, and response to therapeutic interventions. Lancet Diabetes Endocrinol. 2016;4(6):525‐536. 26876794 10.1016/S2213-8587(15)00482-9

[12] Nogueiras R , Nauck MA , Tschöp MH . Gut hormone co‐agonists for the treatment of obesity: from bench to bedside. Nat Metab. 2023;5:933‐944. 37308724 10.1038/s42255-023-00812-z

[13] Santos‐Hernández M , Reimann F , Gribble FM . Cellular mechanisms of incretin hormone secretion. J Mol Endocrinol. 2024;72(4):e230112. 38240302 10.1530/JME-23-0112 PMC10959011

[14] Veprik A , Denwood G , Liu D , et al. Acetyl‐CoA‐carboxylase 1 (ACC1) plays a critical role in glucagon secretion. Commun Biol. 2022;5(1):238. 35304577 10.1038/s42003-022-03170-w PMC8933412

[15] Cyranka M , Veprik A , McKay EJ , et al. Abcc5 knockout mice have lower fat mass and increased levels of circulating GLP‐1. Obesity (Silver Spring). 2019;27(8):1292‐1304. 31338999 10.1002/oby.22521 PMC6658130

[16] Michl J , Park KC , Swietach P . Evidence‐based guidelines for controlling pH in mammalian live‐cell culture systems. Commun Biol. 2019;2:144. 31044169 10.1038/s42003-019-0393-7 PMC6486606

[17] Casey JR , Grinstein S , Orlowski J . Sensors and regulators of intracellular pH. Nat Rev Mol Cell Biol. 2010;11:50‐61. 19997129 10.1038/nrm2820

[18] Cyranka M , Monfeuga T , Vedovato N , et al. NMDA receptor antagonists increase the release of GLP‐1 from gut endocrine cells. Front Pharmacol. 2022;13:861311. 35571112 10.3389/fphar.2022.861311 PMC9091448

[19] Xue JY , Ikegawa S , Guo L . SLC4A2, another gene involved in acid‐base balancing machinery of osteoclasts, causes osteopetrosis. Bone. 2023;167:116603. 36343920 10.1016/j.bone.2022.116603

[20] Michl J , Monterisi S , White B , et al. Acid‐adapted cancer cells alkalinize their cytoplasm by degrading the acid‐loading membrane transporter anion exchanger 2, SLC4A2. Cell Rep. 2023;42(6):112601. 37270778 10.1016/j.celrep.2023.112601

