# Supplementary material for: The release of GLP‐1 from gut L cells is inhibited by low extracellular pH
Source: Obesity (Silver Spring). 2024 Sep 5;32(10):1819–24. doi: 10.1002/oby.24125 (PMC11492159; doi:10.1002/oby.24125)
Supplement: Supplementary file 1 — FIGURE S1: GLP‐1 secretion (pg/mL) from GLUTag cells and primary L‐cells is pH dependent. FIGURE S2: The observed pH effect is not a result of altered ELISA (Cisbio) detection efficiencies. FIGURE S3: Degradation of the secreted GLP‐1 and GLP‐1 peptide stability is not affected by pH. FIGURE S4: Cell viability assays. FIGURE S5: GLP‐1 release rate is pH dependent. FIGURE S6: (A) Representative histograms of pHi measured at pHo 6.4, 7.1 and 7.6 in 10mM glucose. [file OBY-32-1819-s001.pdf]

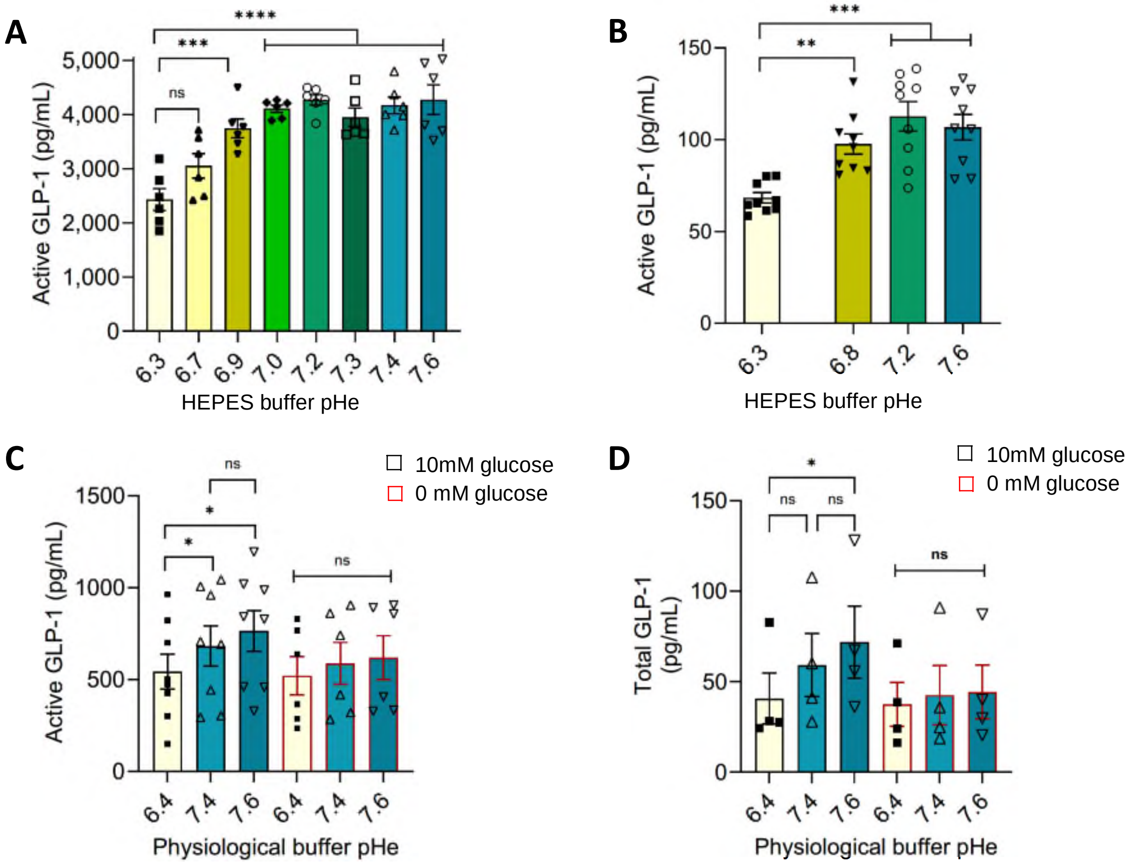

Figure S1. GLP-1 secretion (pg/mL) from GLUTag cells and primary L-cells is pH dependent. **(A)** pH dependence of active GLP-1 secretion (pg/mL) from GLUTag cells in HEPES buffer, 10mM glucose. Data is shown as mean±SEM, n=6. All values recorded for pH6.9 and above are significantly different from pH6.3. Data was generated from a single cell freezer stock using different passage numbers. **(B)** pH dependent secretion of active GLP-1 secretion (pg/mL) from murine mixed primary small intestinal cultures in HEPES buffer. GLP-1 secretion was measured from mixed primary small intestinal cultures isolated from C57BL/6N wt mice. Data is shown as mean±SEM for n=4-5 separate primary culture preparations taken from two different mice. **(C)** pH dependence of active GLP-1 secretion (pg/mL) from GLUTag cells in physiological bicarbonate buffer, 10mM glucose or no glucose. Data is shown as mean±SEM, n=6-8. **(D)** pH dependence of total GLP-1 secretion (pg/mL) from GLUTag cells in physiological bicarbonate buffer, 10mM glucose or no glucose. For C and D data was generated using different freezer stocks and multiple passage numbers. Data is shown as mean±SEM, n=6. *$p<0.05$, **$p<0.01$, ***$p<0.001$, ****$p<0.0001$, ns – not significant. Data for A and B were analysed by one-way ANOVA with multiple comparisons and a Tukey's post-hoc test. Data for C and D were analysed by repeated measure one-way ANOVA with matching, with a Tukey's post hoc test. Matching was statistically significant at $P<0.0001$.

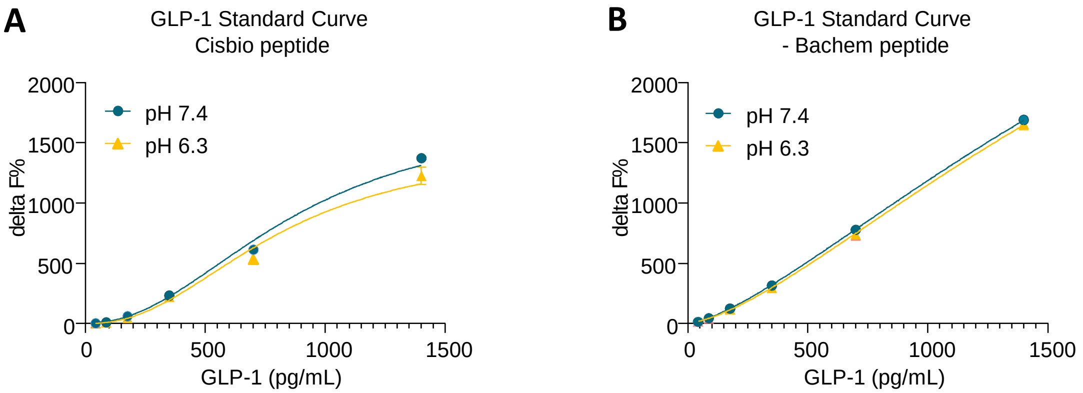

Figure S2. (A) The observed pH effect is not a result of altered ELISA (Cisbio) detection efficiencies. (A) Active GLP-1 standard curves generated by GLP-1 peptide dilution in Krebs buffer at pH6.3 (orange triangles) and pH7.4 (blue circles) using a GLP-1 peptide standard provided by Cisbio at a stock concentration of 56pg/mL. Standard peptide was diluted as per instruction to 1400pg/mL and further two-fold until 43.8pg/. Signal plotted as delta F%, n=2 **(B)** GLP-1 (7-36) amide standard curves generated by peptide dilution in Krebs buffer at pH6.3 (orange triangles) and pH7.4 (blue circles) using a GLP-1 peptide standard provided Bachem at a stock concentration of 300µM diluted to match the concentrations of GLP-1 peptide from (A) in Krebs buffer at pH6.3 and pH7.4. Signal measured plotted as delta F% mean±SD, n=2.

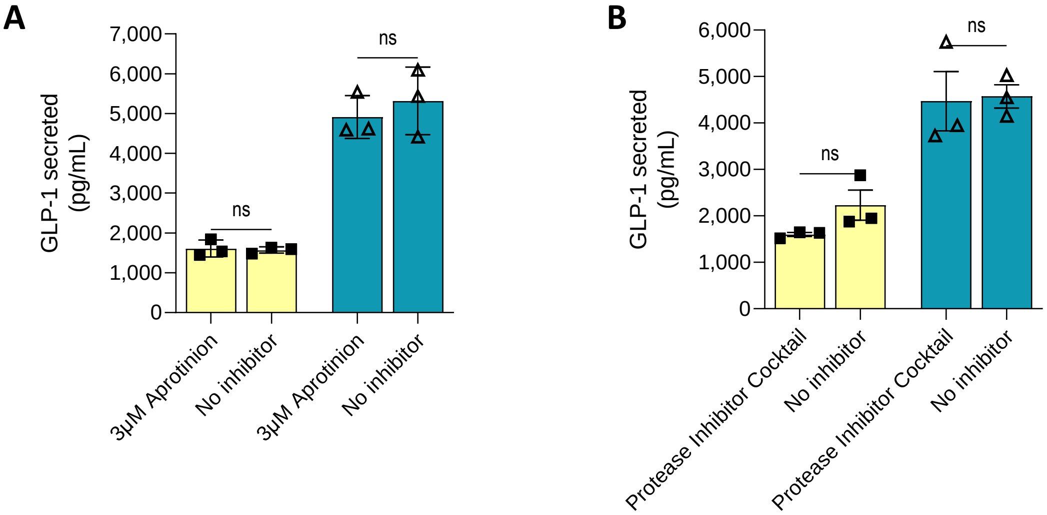

Figure S3. Degradation of the secreted GLP-1 and GLP-1 peptide stability is not affected by pH. (A) GLP-1 secretion (pg/mL) from GLUTag cells after a 2h incubation in the presence of 10mM glucose at pH6.3 (squares) or pH7.4 (open triangles) without protease inhibitor supplementation of the secretion buffer (No inhibitor) or with 3µM Aprotinin (a DPPIV specific inhibitor) **(B)** GLP-1 (pg/mL) secretion from GLUTag cells after a 2h incubation in the presence of 10mM glucose at pH6.3 (squares) or pH7.4 (triangles) without protease inhibitor supplementation of the secretion buffer (no inhibitor) or with commercial protease inhibitor cocktail (PIC) against most common proteases. Data shown as mean±SD for n=3, analysed by two-way ANOVA with Bonferroni's post hoc test, ns – not significant.

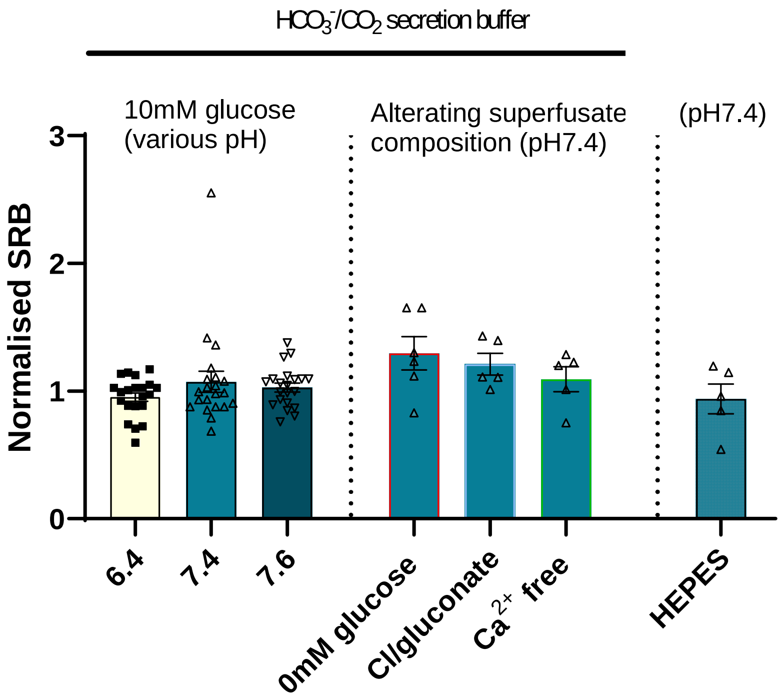

Figure S4. Cell viability assays. Measurements of cell viability of GLUTag cells measured from protein biomass (sulforhodamine B (SRB) assay) at the end of the typical 2h incubation period used in this study. Results are normalized to the average SRB 520nm absorbance of 10mM glucose pH6.4, 7.4, 7.6 measured in parallel on the same day. The statistical comparison was made to pH7.4 standard secretion media (10mM glucose) (one-way ANOVA with Tukey's post hoc test, n=5-21).

Briefly, a sulforhodamine B (SRB) assay was performed after incubation to determine cell density. Cells were fixed using 10% trichloroacetic acid (TCA) at 4°C for at least 60mins. Afterwards, cells were washed with H_2O four times, left to dry, and stained with SRB (0.057% in 1% acetic acid) for 30min. SRB was aspirated, washed four times with 1% acetic acid and left to dry. SRB was then dissolved in 10mM Tris base for 30min. Absorbance was read at 520nm using the Cytation 5 imaging plate reader (Biotek).

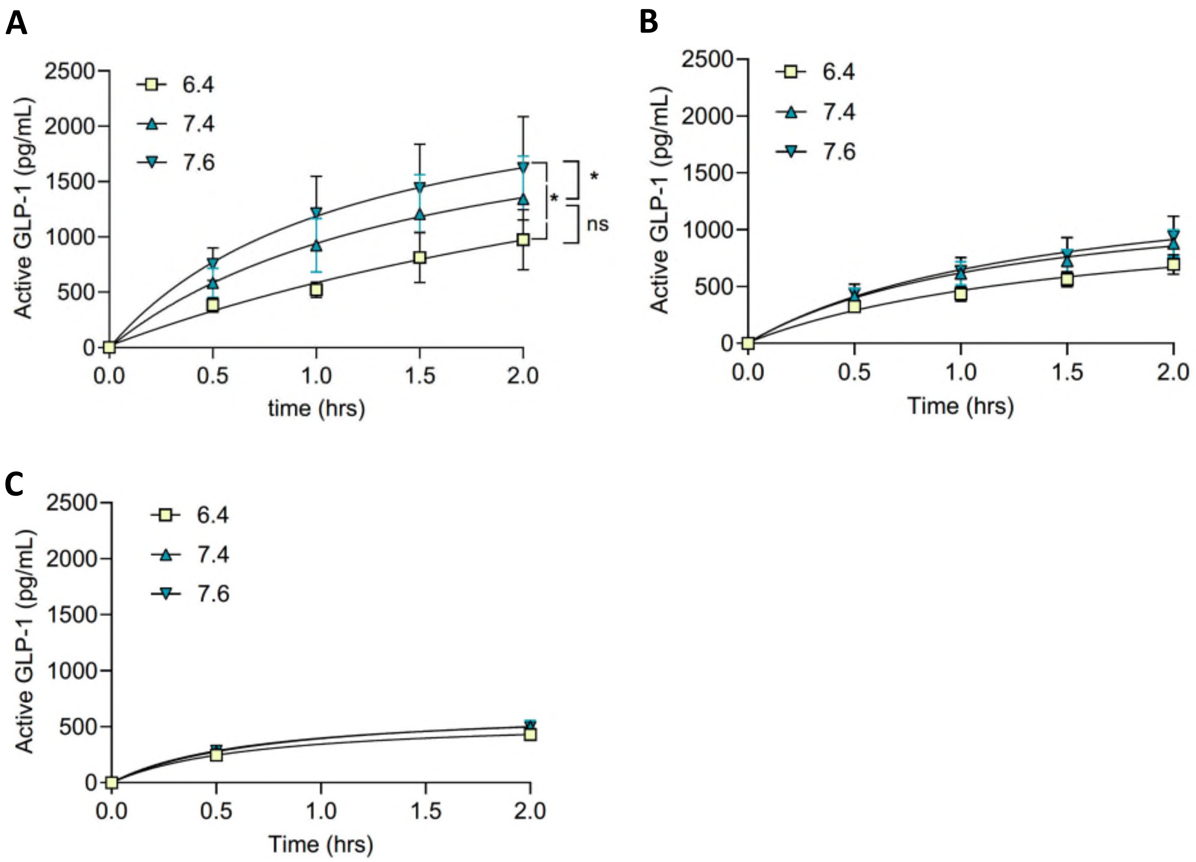

Figure S5. GLP-1 release rate is pH dependent. (A) pHe dependence of the rate of active GLP-1 secretion (pg/mL) from GLUTag cells in physiological buffer in the presence of 10mM glucose, *$p<0.05$ **(B)** no glucose and **(C)** in the absence of Ca^{2+}. The time course of GLP-1 secretion was plotted with the best fit line to the exponential equation $y = a*(1-\exp(-x/b))$. Individual data sets were analysed by repeated measure one-way ANOVA with matching (n=4), with a Tukey's post hoc test. Matching was statistically significant for A at $P<0.0001$.

A

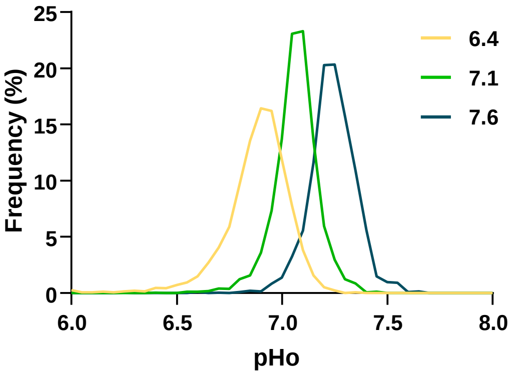

Figure S6. (A) Representative histograms of pHi measured at pHo 6.4, 7.1 and 7.6 in 10mM glucose .
