# Supplementary material for: The release of GLP‐1 from gut L cells is inhibited by low extracellular pH
Source: Obesity (Silver Spring). 2024 Sep 5;32(10):1819–24. doi: 10.1002/oby.24125 (PMC11492159; doi:10.1002/oby.24125)
Supplement: Supplementary file 2 — DATA S1: Supporting Information. Additional supporting information can be found online in the Supporting Information section at the end of this article. [file OBY-32-1819-s002.docx]

**Supplementary Information**

**Methods**

1. **HEPES and CO_2_/HCO_3_^-^ buffered media.**

Medium was made up in solution using deionized water, with added 1% Pen/Strep. For CO_2_/HCO_3_^-^ buffered media (Table S1a), pH was set by adjusting [HCO_3_^-^], achieved by mixing various ratios of stocks containing 44mM NaHCO_3_ (pH7.7) and NaHCO_3_ -free (pH6.2) formulations. Previous work from our laboratory standardised the [HCO_3_^-^] for cell culture medium required to attain a target pH at 5% CO_2_ and the concentration of [HCO_3_^-^] mM at 5% CO_2_ from pH6.3 to pH7.6 is detailed in Table S1b [16]. To account for differences in osmolarity, 44mM NaCl or gluconate (NaC_6_H_11_O_7_) was added to 0mM HCO_3_^-^ solutions. For the latter, increases in CaCl_2_ were necessary to compensate for gluconate interactions (chelating) with Ca^2+^ in solution. Alternatively, cells were treated with HEPES buffered media (Table S2) and pH adjusted with NaOH, accurately determined using a pH meter at 37°C. In HEPES buffered media, cells were incubated at 37°C.

Glucose was the primary secretagogue used to stimulate GLP-1 secretion. ‘Standard secretion media’ refers to the CO_2_/HCO_3_^-^ buffered solution with added osmolarity regulating NaCl, containing 10mM glucose. Additives were added to the media as indicated in the legends to the figures and text. The following volumes of secretion buffer were used: 0.25ml/well for 24-well plates (GLP-1 secretion assays) or 0.2ml/well for 96-well plates (SNARF based assays).

For all experiments, cells were counted electronically using the Life Technologies Countess II Automated Cell Counter and re-seeded to the desired density into cell culture dishes coated with Poly-L-lysin (P4707-50mL, Sigma). In 96-well plates, cells were typically seeded at 1.5 x 10^5 cells/well, in 24-well plates at 3 x 10^5 cells/well and cells were allowed to grow for 48hr-72hr to achieve 70%< confluency.

**Table S1a. Composition of CO_2_/HCO_3_^−^ based stock medium.**

| a | 0mM glucose | | | 10 mM glucose | | | |
| --- | --- | --- | --- | --- | --- | --- | --- |
| Ingredients | pH7.7  (mM) | pH6.2 (mM) | pH6.2  (Gluconate) (mM) | pH7.7  (mM) | pH6.2  (mM) | pH6.2  (Gluconate) (mM) | pH7.4  Ca^2+^ free  (mM) |
| CaCl_2_ | 1.8 | 1.8 | 3.188 | 1.8 | 1.8 | 3.188 | - |
| Fe(NO_3_)_3_ • 9H_2_O | 0.248 | 0.248 | 0.248 | 0.248 | 0.248 | 0.248 | 0.248 |
| MgSO_4_ | 0.811 | 0.811 | 1.003 | 0.811 | 0.811 | 1.003 | 0.811 |
| KCl | 5.4 | 5.4 | 5.4 | 5.4 | 5.4 | 5.4 | 5.4 |
| NaCl | 117.5 | 161.5 | 117.5 | 117.5 | 161.5 | 117.5 | 139.5 |
| NaH_2_PO_4_ | 0.908 | 0.908 | 0.908 | 0.908 | 0.908 | 0.908 | 0.908 |
| D-Glucose |  | - | - | 10 | 10 | 10 | 10 |
| NaHCO_3_ | 44 | - | - | 44 | - | - | 22 |
| NaC_6_H_11_O_7_  Sodium gluconate | - | - | 44 | - | - | 44 | - |
| EGTA | - | - | - | - | - | - | 0.5 |

**Table S1b. [HCO_3_^-^] required for target pH in CO_2_/HCO_3_^−^based medium.**

| Target medium pH | [HCO_3_^-^] mM at 5% CO_2_ |
| --- | --- |
| 6.3 | 1.65 |
| 6.5 | 2.75 |
| 6.8 | 5.5 |
| 7.1 | 11 |
| 7.4 | 22 |
| 7.6 | 33 |

**Table S2. Composition of HEPES based medium used in Fig.1A and B. pH was titrated to the desired value using 5N NaOH at 37°C and 0% CO_2_.**

|  | 0mM glucose | 10mM glucose |
| --- | --- | --- |
| Ingredients | pH7.4  (mM) | pH7.4  (mM) |
| NaCl | 135 | 140 |
| HEPES | 20 | 20 |
| KCl | 4.5 | 4.5 |
| MgCl_2_ | 1 | 1 |
| CaCl2 | 1 | 1 |
| D-Glucose | - | 10 |

**Table S3. Composition of HEPES based medium used in Fig.3A. pH was titrated to the desired value using 5N NaOH at 37°C and 0% CO_2_.**

| Ingredients |  |
| --- | --- |
| CaCl_2_ | 1.8 |
| Fe(NO_3_)_3_ • 9H_2_O | 0.248 |
| MgSO_4_ | 0.811 |
| KCl | 5.4 |
| NaCl | 151.5 |
| NaH_2_PO_4_ | 0.908 |
| HEPES | 20 |
| D-Glucose | 10 |
| NaHCO_3_ | - |
| NaC_6_H_11_O_7_  Sodium gluconate | - |
| EGTA | - |

1. **pHi measurements**

Intracellular pH was measured using carboxy SNARF-1 (cSNARF1) in cells identified by particle analysis of fluorescence centred around nuclei visualized using the DNA-binding stain Hoechst-33342. In black wall, flat coverslip bottom µ-plate 96-well plates, cells were seeded in triplicated at 1.5 x 10^5^ cells/well and grown for 48hr. For experimentation, growth media was aspirated and replenished with 0.2ml/well pH specific bicarbonate or HEPES media. After a 2h incubation period, cells were then incubated in media supplemented with cSNARF1-AM (5 mg/mL, Molecular Probes) and the nuclear stain Hoechst-33342 (10 mg/mL, Molecular Probes), for 15mins. Media was aspirated, washed twice and replenished with 0.2ml/well pre-incubated pH specific medium. Plates were imaged using the Cytation 5 imaging plate reader (Biotek) and measurements performed in an atmosphere of 37°C, and either 0% CO_2_ in HEPES or 5% CO_2_ in bicarbonate buffer. Hoechst-33342 fluorescence was detected by 377nm excitation and 447nm emission, and cSNARF1 fluorescence excited by 531nm and simultaneously collected at 590nm and 640nm. Further analysis of the population distribution of pH data was performed with a MATLAB script139 (Figure S6).

**3.** **Cell viability analysis using SRB**

To confirm cell viability was not compromised by condition-specific media, a sulforhodamine B (SRB) assay was taken after incubation to determine cell density. Cells were fixed using 10% trichloroacetic acid (TCA) at 4°C for at least 60mins. Afterwards, cells were washed with H_2_O four times, left to dry, and stained with SRB (0.057% in 1% acetic acid) for 30min. SRB was aspirated, washed four times with 1% acetic acid and left to dry. SRB was then dissolved in 10mM Tris base for 30min. Absorbance was read at 520nm using the Cytation 5 imaging plate reader (Biotek).

4. **Glucagon-like peptide 1 (GLP-1) secretion**

To quantify GLP-1 secretion, supernatant samples were taken from GLUTag cell cultures seeded in dishes coated with Poly-L-Lysin and grown for 48hr at 37°C, 5% CO_2_. After incubation, media was aspirated and replaced with experimental specific secretion buffer (Table S1a). Supernatant samples were taken at the 2hr end-point, or where the rate of GLP-1 secretion was investigated, supernatant samples were collected every 30mins for up to 2hr and replenished with fresh media to maintain volume of the incubating media. In each assay experiment, 100µL of supernatant sample was collected from each well. For GLP-1 degradation rate studies (Figure S2), 0.5ml supernatant samples were collected from GLUTag cells incubated in 1ml bicarbonate buffered media for 1hr. Supernatant was stored in 24-well plates, and every 30mins, 0.05ml samples collected for quantification.

Samples were collected in Eppendorf tubes pre-loaded with a mixture of detergents of 0.5% Triton-X and 0.4% Tween-20 (w/v) in ultrapure water. This was diluted 10-fold with the secretion supernatant. This approach was suggested to avoid GLP-1 sticking on tubes and tips and maximize recovery of GLP-1 for future measurements. Harvested samples were stored on ice until all samples were collected. After this point, Eppendorf tubes were spun at 4°C, 300xg for 5mins to remove floating cells and debris, 0.05ml supernatant harvested into fresh Eppendorf tubes and stored at -80°C until further processing, or supernatant was used immediately for [GLP-1] quantification. The remaining cell monolayer was washed with DPBS and fixed with 10% TCA for SRB protein quantification.

**5. Glucagon-like peptide 1 (GLP-1) detection**

The amounts of active GLP-1 (7-37 and 7-36-NH_2_) secreted by GLUTag cells were measured in secretion medium by FRET-based HTRF assay (Active GLP-1, Cisbio) according to manufacturer’s protocols. Final concentrations of secreted GLP-1 were calculated from the standard curve generated by active GLP-1 peptide standard provided by the manufacturer and presented as pg/mL. GLP-1 stock solution at 2800pg/mL was prepared in ultrapure water and serial dilutions were made in experimental secretion buffer. Where stated, the Multi Species GLP-1 Total ELISA kit was also used to measure and quantify total (active plus inactive) supernatant [amidated GLP-1] according to manufacturer’s protocols (catalog #EZGLP1T‐36K; Millipore, Burlington, MS).
